# Successful Use of Ultrasound Guided Quadratus Lumborum Block Without General Anesthesia for Open Appendectomy in a Patient with Heart Failure with Reduced Ejection Fraction- A Case Report and Literature Review

**DOI:** 10.2147/LRA.S438176

**Published:** 2024-02-23

**Authors:** Muhammad Jaffar Khan, Yasir Eltayeb, Arunabha Karmakar, Rohma Malik, Tarig Elsafi

**Affiliations:** 1 Department of Anesthesiology, Critical Care and Perioperative Medicine, Hamad Medical Corporation, Doha, Qatar

**Keywords:** quadratus lumborum block, regional anesthesia, heart failure with reduced ejection fraction, left ventricular ejection fraction, quadratus lumborum muscle, appendectomy

## Abstract

**Background:**

Patients diagnosed with Heart Failure with Reduced Ejection Fraction (HFrEF) are at high risk of perioperative cardiovascular complications. While it is important to focus on optimizing their cardiac function, it is also crucial to address and optimize any other modifiable risk factors that could potentially impact postoperative outcome. This also includes careful consideration of anesthetic techniques to suit the patient and facilitate the surgery. However, there is a scarcity of evidence regarding the safety of specific anesthetic approaches for heart failure patients.

**Case Presentation:**

We describe the case of an adult patient in mid-50s, with a history of ischemic dilated cardiomyopathy with reduced Ejection Fraction (about 25%) who presented with acute gangrenous appendicitis and was scheduled for an open appendectomy. It was deemed to be a high-risk patient for general and spinal anesthesia. With the guidance of a multidisciplinary team, surgery was successfully performed using a quadratus lumborum block with standard monitoring. The patient was comfortable and hemodynamically stable throughout the procedure. The postoperative course was uneventful.

**Conclusion:**

Quadratus Lumborum Block for open appendectomy can be a beneficial alternative anesthesia technique in high-risk patients that significantly lowers perioperative cardiovascular risk, maintains hemodynamics, enhances satisfaction, and shortens hospital stay.

## Introduction

Patients with Heart failure with reduced Ejection Fraction (HFrEF) pose significant perioperative challenges due to risk of developing perioperative major adverse cardiovascular events (MACE). These patients need optimization of preload, afterload reduction, and minimization of myocardial depression to maintain cardiac output.^1^ Therefore, anesthetic techniques should be instituted wisely to prevent perioperative morbidity and mortality.^2^,^3^

For high-risk patients requiring surgery, safer options such as neuraxial or peripheral nerve blocks are advisable. Modern ultrasound imaging aids in precise and targeted local anesthesia, enabling surgeries without compromising vital organ function. We report our early success using a Quadratus Lumborum Block (QLB) for near-complete anesthesia in open appendectomy. Although the QLB is well-known for postoperative pain relief, its use as the sole anesthetic technique in abdominal surgery has not yet been well-substantiated.

With adequate monitoring and a careful approach, we report a successful case of appendectomy performed under QLB in a patient with heart failure with reduced ejection fraction (HFrEF).

## Case Report

A 56-year-old gentleman (65 kg, 160 cm, BMI 25.4 kg/sq.m and ASA-IV E) presented to our emergency department with a two-day history of right lower abdominal pain, fever, and vomiting. His medical history was significant for long-standing type 2 diabetes mellitus (DM), hypertension, dyslipidemia, and dilated cardiomyopathy. Examination revealed tenderness in the right iliac fossa and bilateral fine basal crepitation. CT abdomen revealed acute gangrenous appendicitis, and an urgent open appendectomy was scheduled.

### Preoperative Assessment and Optimization

We assessed him preoperatively and consulted with a cardiologist. He was admitted to the Emergency Department, and our focus was on the patient’s cardiorespiratory system and ability to tolerate a general anesthetic procedure.

On examination, he was stable enough to verbalize his medical history. He also had a history of hospital admission for decompensated heart failure in 2019. At that time, transthoracic echocardiography (TTE) revealed a dilated left ventricle (LV), severely reduced systolic LV function (EF 18%), severe global hypokinesis of LV, and Grade 3 diastolic dysfunction. The patient was treated with anti-heart failure medications and discharged home a week later. He was prescribed aspirin, bisoprolol, ramipril, dapagliflozin, rosuvastatin, isosorbide dinitrate, and insulin.

In this visit, he had poorly controlled DM (HbA1C 13%). Laboratory tests revealed an NT pro-BNP level of 1122 pg/mL, troponin T level of 28 ng/l, CRP level of 158 mg/l, WBC 16000/mcl. TTE revealed a moderately dilated left ventricle, severely reduced LV systolic function, biplane LVEF of approximately 25%, severe global hypokinesis of the LV, and Grade 3 diastolic dysfunction. His medications on admission included bisoprolol, rosuvastatin, and insulin.

The patient had a revised cardiac risk index (RCRI) of 4, with 15% risk of major adverse cardiac events (MACE) and high risk for both general anesthesia and neuraxial blockade.

He was admitted to the SICU for preoperative optimization, arterial and central venous line insertion, Pulse index Continuous Cardiac Output (PiCCO)-guided fluid resuscitation, and antibiotic prophylaxis.

#### Multi-Disciplinary Team Meeting

Owing to the patient’s high risk, we held a multidisciplinary team meeting. The team decided that the patient would undergo an open appendectomy with QLB combined with monitored anesthesia care (MAC). As the patient had gangrenous appendicitis, there was a further risk of deterioration.

#### Intraoperative Management

The patient was counselled regarding the urgent need for surgery, perioperative risk of cardiac complications, and specific anesthesia management, and written consent for anesthesia was obtained. He was brought to the operating room, and standard monitors were applied, along with an invasive arterial line monitor and supplemental oxygen administered via face mask at 5L/min. Inotropes (dobutamine and adrenaline infusions) were prepared in case he developed hemodynamic instability.

**Figure 1:**
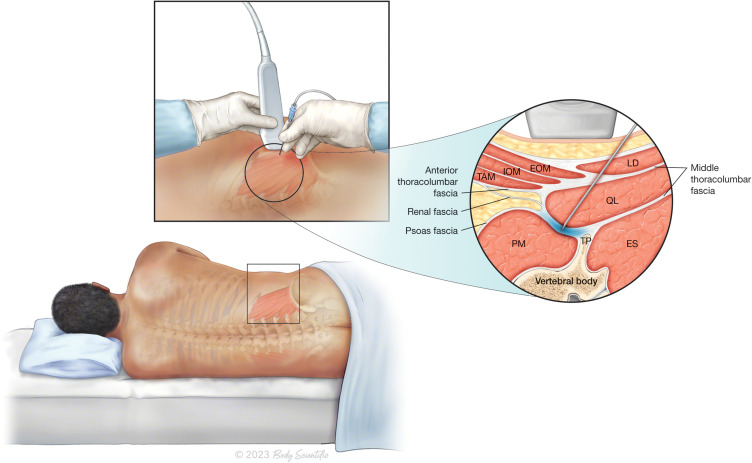
Anterior approach to a Quadratus Lumborum Block. Local anaesthesia is injected under ultrasound guidance and aseptic precautions to a space between the Psoas Major Muscle and Quadratus Lumborum Muscle (Abbreviations EOM: External Oblique Muscle; IOM: Internal Oblique Muscle; TAM: Transversus Abdominis Muscle; PM: Psoas Major Muscle; ES: Erector Spinae Muscle; QL: Quadratus Lumborum Muscle; LD: Latissimus Dorsi Muscle; TP: Vertebral Body Transverse Process). ©2023 Body Scientific International, LLC.

In the left lateral decubitus position, 2 mg of midazolam and 50 µg of fentanyl were administered intravenously as anxiolytics. An ultrasound probe (curvilinear low-frequency) was placed in a transverse orientation at the midaxillary line at the L2-L4 level to visualize the three expected abdominal layers (transversus abdominis, external oblique, and internal oblique). The probe was then moved posteriorly until quadratus lumborum muscle (QLM) was confirmed. Under aseptic techniques and ultrasound guidance, the needle was inserted and advanced into the anterior aspect of the QLM. Proper positioning of the needle tip was confirmed by hydrodissection, and 20 mL of 0.33% levobupivacaine was injected into the fascia between the right QL muscle and psoas muscles (Figure 1).

Surgery was performed 15 min after the block, with supplemental local infiltration and intravenous fentanyl at titrated doses of up to 50 µg. Intraoperative sedation was achieved with 2 mg of midazolam intravenously at titrated doses of up to 4 mg. A minimal dose of noradrenaline 0.03–0.05mcg/kg/min infusion was continued to maintain normal hemodynamics. An additional dose of intravenous fentanyl (50 µg) was administered to blunt the effects of deep peritoneal stimulation. The patient maintained the airway and vital signs around baseline throughout the procedure.

#### Postoperative Management

**Figure 2:**
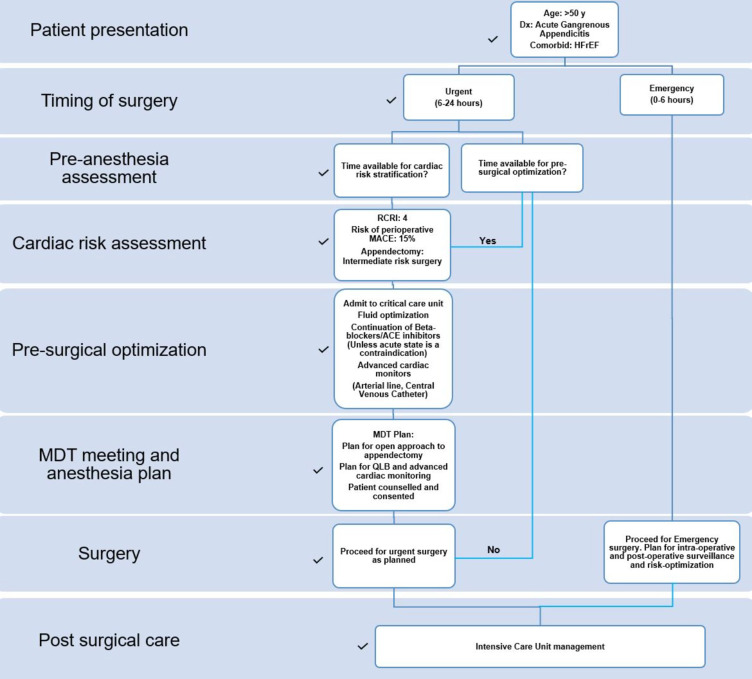
Clinical care pathway for our patient taking into consideration guidelines and recommendations for management of a cardiac patient presenting for non-cardiac surgery from the American Heart Association, American College of Cardiology, European Society of Cardiology and Canadian Cardiovascular Society. Additionally, we used input from a multidisciplinary team meeting with the surgical team and senior anaesthetists to make our decision. The black ticks highlight our steps in each phase of the patient’s care.

**Notes**: Data from these studies.^3–7^

**Abbreviations**: HFrEF, Heart Failure with Reduced Ejection Fraction; RCRI, Revised Cardiac Risk Index; MACE, Major Adverse Cardiac Events; ACE Inhibitors, Angiotensin Converting Enzyme Inhibitors; MDT, Multidisciplinary Team; QLB, Quadratus Lumborum Block.

The patient was transferred to the SICU for hemodynamic monitoring and organ support. The numerical rating of pain score was 0/10 at rest and 2/10 with movement at 24 h and reached a maximum of 4/10 with movement at 48 h postoperatively. His pain was managed with paracetamol, and he was discharged on the fourth day postoperatively. A summary of the clinical care pathways for our patient is shown in Figure 2.

**Figure 3:**
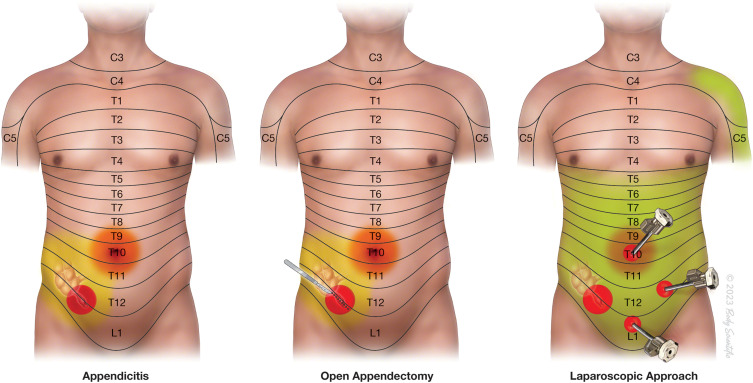
Pain type, intensity and distribution in Appendicitis patients. Acute appendicitis (top left) mainly produces well-localized sharp pain (red color overlay) in the left lower abdomen, with referred pain to the umbilicus (yellow color overlay), which is more poorly localized. Open appendectomy (top middle) produces sharp incisional pain in addition to pain associated with acute appendicitis (red overlay). Further pain occurs during blunt dissection of deeper muscles and tissues, and visceral pain occurs during peritoneal incision and appendix manipulation. Laparoscopic appendectomy (top right) produces sharp incisional pain at the sites of trocar insertion in addition to the pre-existing pain of acute appendicitis (red overlay). After carbon dioxide insufflation, diffuse stretching of the abdomen and peritoneum results in diffuse and poorly localized discomfort (green overlay). There is also referred pain in the shoulder due to irritation of the diaphragm (green overlay). ©2023 Body Scientific International, LLC.

**Figure 4:**
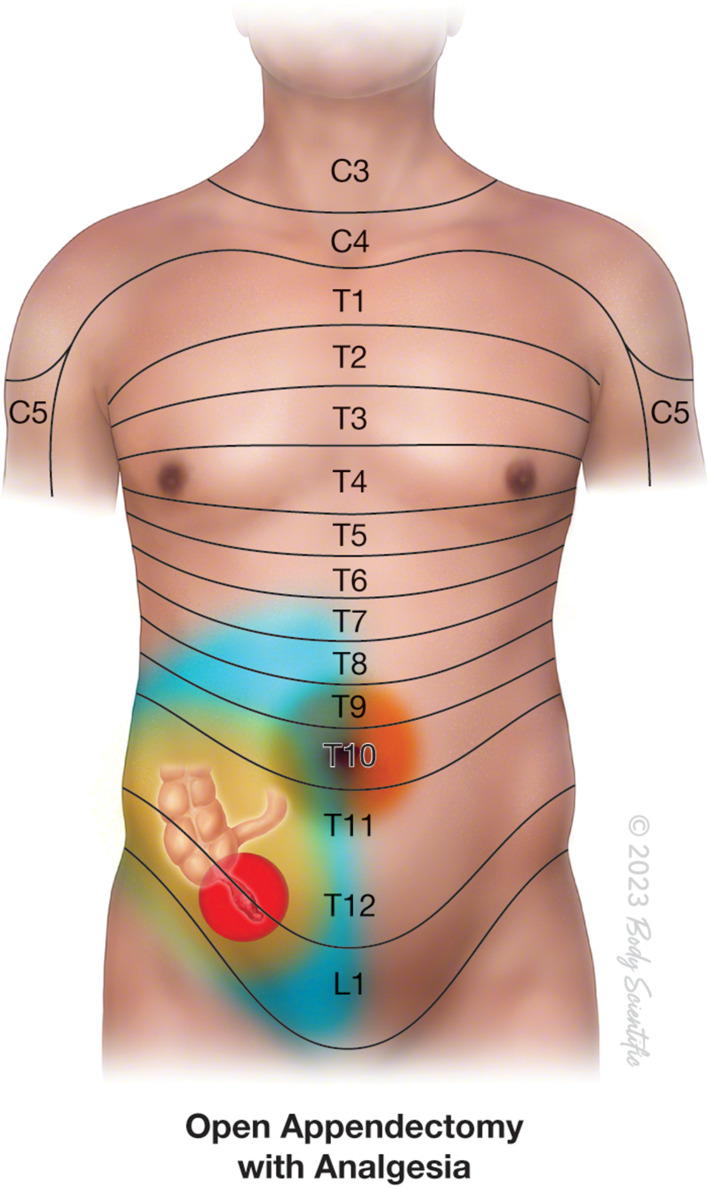
Anesthetic coverage by a well performed Quadratus Lumborum block providing adequate coverage of the pain of open appendectomy (blue overlay). However, some amount of visceral pain is not completely blocked. ©2023 Body Scientific International, LLC.

## Discussion

### Heart Failure and Perioperative Cardiac Risk

The worldwide incidence of heart failure is approximately 64 million.^8^,^9^ HFrEF is a global health concern and poses many anesthetic challenges.

HFrEF is associated with significant perioperative morbidity and mortality.^2^,^8^,^10^,^11^ While in-hospital mortality of HFrEF is greater than that of HFpEF (heart failure with preserved ejection fraction), there are no clinically significant differences in mortality at 30 days or up to 1 year and no clinically significant differences in hospital readmission rates.^8^,^12^

Key to successful anesthetic management in patients with HFrEF includes detailed knowledge of pathophysiological changes, precise intraoperative monitoring, intraoperative modulation of hemodynamic parameters, the right choice of anesthetics, and multidisciplinary team input.^1^

#### Benefits of Regional Anaesthesia in Heart Failure

General anesthesia and neuraxial anesthesia can reduce cardiac output via the loss of sympathetic tone during induction. This can cause life-threatening circulatory collapse in heart failure patients. It is beneficial to offer locoregional anesthesia to patients undergoing minor or peripheral procedures when possible to preserve cardiac output and minimize myocardial work.

Patients with HFrEF are particularly sensitive to tachycardia, which leads to increased myocardial oxygen demand. Etiologies leading to tachycardia, such as intubation, surgical stimulation, postoperative pain, nausea, and vomiting, are therefore best avoided and mitigated. Intubation is avoided when possible by choosing adequate locoregional anesthesia, and surgical stimulus and postoperative pain are both mitigated and/or abolished. In addition, the patient’s cough reflex remains intact and is thus able to protect his or her airway. In heart failure with reduced ejection fraction (HFrEF), left ventricle filling relies on both atrial contraction and the pressure gradient between the left atrium and the left ventricle. A slight decrease in preload results in a swift reduction in stroke volume (SV), and thus hypovolemia is poorly tolerated. However, even a minor volume load or an increase in afterload can lead to rapid decompensation of the heart, attributed to elevated left ventricular end-diastolic pressure (LVEDP). Therefore, achieving a fine equilibrium between preload and afterload is crucial for improving outcomes and preventing perioperative complications.

By reducing opioid consumption and volatile anesthetics, major risk factors for postoperative nausea and vomiting are also avoided.^13^

Other potential benefits include better perioperative pain control than intravenous opioids, potentially reduced blood loss, reduced perioperative rates of deep vein thrombosis, reduced postoperative fatigue or confusion, earlier bowel function recovery, earlier discharge from the recovery room and hospital, and earlier ambulation and physical therapy.

#### Quadratus Lumborum Block and Appendectomy

For effective regional anaesthesia, it is important to isolate the nociceptive pathways that are involved in a patient with acute appendicitis undergoing appendectomy (Figure 3). The incision of open appendectomy traverses dermatomes innervated by the right Iliohypogastric (L1) and Ilioinguinal nerve (L1) and occasionally the 12th intercostal nerve. In Ultrasound-guided QLB, a local anesthetic is administered adjacent to the quadratus lumborum (QL) muscles to anesthetize the thoracolumbar nerves. Many published studies support the role of the QLB as the main component of multimodal analgesia for caesarean section deliveries and urologic or abdominal surgeries.^14^ Few studies have demonstrated the successful use of the QLB (Table 1) as a solitary anesthetic technique in abdominal surgeries.^15–17^ However, there is a paucity of evidence regarding its use as the primary surgical anesthetic technique, especially for appendectomy.

**Table 1:** Summary of Case Reports of Quadratus Lumborum Block as Anesthetic Technique for Abdominal Surgeries

Case Type	Surgery	Comorbid Conditions	Quadratus Lumborum Approach	njectate	Additive Sedation/Analgesics	Postoperative Analgesia
Cassai et al^18^	Left inguinal incarcerated hernia repair	Dilated cardiomyopathy with EF 33%, CAD, pacemaker implantation for a third degree AV block, severe pulmonary HTN, severe mitral valve insufficiency and third stage CKD	Injectate given at the interfascial plane between the QL muscle and psoas muscle	30 mL of 0.5% ropivacaine	Conscious sedation with propofol 1mg/kg/hr and remifentanil 0/05 mcg/kg/min with additional local anesthetic infiltration with lidocaine 2% during manipulation of the hernia sac	Postoperative analgesia with paracetamol only and discharged on the fourth postoperative day
La Colla et al^15^	Right inguinal hernia open repair	Multiple system Atrophy, OSA, Autonomic dysfunction with severe orthostatic hypotension	Injectate given at the interfascial plane between the QL muscle and psoas muscle	20 mL of 0.5% ropivacaine	Fentanyl 50mcg i.v before block Sedation with low-dose propofol (10mcg/kg/min). Local infiltration with 6mL of 0.25% bupivacaine prior to incision	Postoperative analgesia with paracetamol and pregabalin 100 mg overnight
Balogh et al^16^	Open umbilical Hernia repair (case 1)	CM with EF of < 10%, CAD, end stage liver disease and CKD	Injectate given bilaterally into the lateral interfascial triangle behind QL muscle (QLB type 2)	20 mL of 0.5% ropivacaine	1 mg of i.v midazolam prior to block followed by 0.1 mcg/kg/min of remifentanil infusion	Multimodal modal analgesia (acetaminophen, gabapentin and tramadol)
Balogh et al^16^	Open repair of incarcerated ventral hernia (case 2)	CM with an EF< 10%, CAD, COPD, End stage renal disease, HTN, history of cocaine abuse	Injectate given bilaterally into the lateral interfascial triangle behind QL muscle (QLB type 2)	20 mL of 0.5% ropivacaine	Sedation with 0.1 mcg/kg/min infusion of remifentanil and 0.04 mcg/kg/hr of dexmedetomidine	Not reported
Mohan et al^19^	Open repair of left inguinal hernia repair	CAD with severe left ventricular dysfunction, DM-II, CLD	Injectate given between Quadratus lumborum and psoas muscles. Indwelling catheter inserted	25 mL of 0.5% ropivacaine	None	Ropivacaine (0.125%) 5mL/hr via indwelling catheter
Vieira et al^17^	Terminal colostomy for enterovesical fistula	CAD with a previous MI, chronic anemia, HTN, CKD, DM-II	Injectate deposited anterolateral to the QL muscle, lateral to the transversus abdominis muscle	20 mL of 1.33% of Mepivacaine	4 mg of dexamethasone i.v and fractionated boluses of fentanyl to a total of 75 mcg i.v given	No postoperative analgesia required

**Abbreviations**: CAD, coronary artery disease; EF, ejection fraction; AV, atrioventricular; HTN, hypertension; IV, intravenous; CKD, chronic kidney disease; QL, quadratus lumborum; OSA, obstructive sleep apnea; CM, cardiomyopathy; QLB, quadratus lumborum block; COPD, chronic obstructive pulmonary disease; DM-II, diabetes type 2; CLD, chronic liver disease; MI, myocardial infarction.

Detailed knowledge of the anatomy and relevant technical aspects of the quadratus lumborum block (QLB) is important for its successful and effective use. The QLM is a posterior abdominal wall muscle that arises from the posteromedial iliac crest and inserts into the transverse processes of the L1 to L4 vertebrae and the medial border of twelfth rib. Dermatomal sensory blockade from T12 to L2, including the iliohypogastric and ilioinguinal nerves, has been demonstrated.^20^ Three different approaches to quadratus lumborum block have been suggested, which are based on the anatomical location of the needle and local anesthetics injected into the quadratus lumborum muscle. This is thus termed anterior, lateral, and posterior techniques. In the anterior approach, the point of injection of the local anesthetic lies in the interfascial plane between the quadratus lumborum and the psoas muscle, whereas in the lateral technique, the injectate is administered along the lateral border of the quadratus lumborum muscle. Finally, for the Posterior approach, the local anesthetic is administered at the posterior border of the quadratus lumborum between the QL and erector spinae muscles. In addition to the thoracic paravertebral spread of local anesthetic, the injectate may spread to the lumbar plexus and its branches in the anterior QLB as demonstrated in many cadaveric and case studies.^21–23^

Given the substantial cardiovascular risk factors as well as the multiple comorbid conditions in our patient, we chose to perform QLB using the anterior approach as the primary anesthetic technique (Figure 4). The patient was counselled about the innovative nature of the anesthesia technique that would be utilized. This comprehensive discussion aimed to ensure the patient’s understanding and informed decision-making regarding the impending procedure, potential challenges, and the unique aspects of the anesthesia approach. General anesthesia and neuraxial techniques were avoided in this patient because of their propensity to develop systemic hypotension and myocardial depression. We opted for the quadratus lumborum block because it provides superior analgesia compared with the transversus abdominis plane block and offers increased dermatomal coverage and visceral analgesia.^14^,^24^ Alternatively, the Erector spinae plane block at the level of the transverse process (T8) has been suggested as the main component of multimodal analgesia in abdominal surgeries.^25^,^26^ It can also provide visceral analgesia through the spread of local anesthesia to the paravertebral space. However, its efficacy as the sole anesthetic for abdominal surgeries has yet to be validated.

## Conclusion

This case describes the successful utilization of an innovative anesthesia technique (Quadratus Lumborum Block) for urgent appendectomy in a high-risk patient with significant cardiovascular morbidity, thus reducing the perioperative risk of major cardiovascular events, maintaining perioperative hemodynamic status, excellent patient satisfaction, and reducing hospital stay.
